# Clinical Notes

**Published:** 1875-07

**Authors:** F. K. Bailey

**Affiliations:** Knoxville, Tenn.


### CLINICAL NOTES.

BY F. K. BAILEY, M.D., KNOXVILLE, TENN.

### *NAUSEA OF PREGNANCY.*

January 30th, 1875.—Mrs. ——, a well-formed and very intelligent lady ; æt. about 30 ; nervous, bilious temperament ; has three healthy children ; has at every conception begun at once to suffer from intense nausea, with more or less vomiting of food ; has missed three weeks, and suffered for the last ten or twelve days. Since the 27th inst. she has been confined to her bed. Saw her at

7 P.M., and found a slow, weak pulse, paleness of the cutaneous surface, and an extremely anxious countenance. Tongue not coated, but looking rather pale and flabby. Is constantly sick at the stomach, and cannot retain food or drinks. There is a constant and increased amount of salivary secretion pouring into the mouth which tastes very badly, and adds to the nausea. Bowels inclined to constipation. No natural appetite or unnatural "longings." Urine red, but not scanty, and is passed easily. Prescribed as follows: ℞. Oxalate of cerium, grs. viij.; sub. nit. bismuth, grs. xx. M. F. pulv. No. 8. Sig. One every four hours, in dry sugar.

For a pain complained of in the chest at the middle of the sternum, ordered a belladonna plaster applied at once. This pain is attributed to exhaustion from nausea.

31st, 9 A.M.—Feeling some better. Nausea continues, but retains food. To continue powders, with small quantities of whisky toddy. 5 P.M. Still better, but nausea not entirely relieved. Prescribed—℞. Bromide potassium, hydrate chloral, aa. ʒi.; glycerin, ℥ss.; aquæ amygdal amavæ, ℥iss. M. F. enema. Sig. One teaspoonful in ℥ij. tepid water, introduced per rectum, every two or three hours; continue powders.

February 1st, 9 A.M.—Used the enema once only, and found almost immediate relief. There was a sense of quiet felt, so that she could sleep more than had been enjoyed for some days. Can retain food with a relish, and there is an increase of vascular force shown by a stronger and fuller pulse. No change in the salivary secretion. To repeat the enema in double the amount should nausea recur. To take soups and whisky punch with cracker soaked in it. Must avoid seeing company, or talking with the family, so far as possible.

2d, 10 A.M.—Has suffered but little from nausea for the last twenty-four hours, and has some appetite for food. Complains at times of a fluttering at the heart, and slight dyspnœa, which may be attributed to debility. She used the enema but once since yesterday morning, and experienced relief. To continue the enemata twice in the day, and take moderately of toddy; also the following pill once or twice daily: ℞. Ext. belladonna, grs. iij.; oxalate of cerium, grs. xij.; ext. savaxæum, q. s. M. F. pil. No. 12.

3d.—Slept badly in consequence of fatigue from talking with lady visitors. Vomited once since yesterday, but retains food. The secretion from salivary glands is decreasing. Pulse fuller, but still weak. Bowels moved slightly by enemata. The sedative enema used last night produced some quiet. To continue treatment.

4th, 11 A.M.—Vomited once this morning on raising up, but threw up only water and mucus. Disturbed last night by a sick child, but slept some. Appetite good, and retains food. Bowels open once. Some drivelling of saliva. No dryness in the throat. Improving a little in strength. To have enemata of warm water, and also the bromide and chloral mixture. Continue the pills. Avoid exertion, and take toddy as indicated.

5th, 11 A.M.—Sitting up in her easy chair. Has felt little or nothing of nausea since yesterday, and can eat with a relish. The salivary secretion much lessened, but there is a great perversion of the sense of taste. She desires articles to which she is always indifferent when not pregnant, and loathes what was most acceptable. She also desires pickles which are prepared with pepper, and can eat pepper in every form without appreciating the pungency which is otherwise very noticeable. This affection of the gustatory apparatus has always obtained in former pregnancies in connection with other symptoms. To continue the enema at night, and the pills as before; toddy *ad libitum.*

6th.—Vomited last night after eating some oysters. She has never been able heretofore to retain them. Otherwise no worse. Treatment continued.

7th.—Has sat up all day in a chair. Vomited slightly on first raising up in bed in the morning. Retained food and feels very well, except that she is weak. The bowels are torpid, and warm water enemata have but little effect. Takes the oxalate cerium pills. Omitted the bromide enema for two nights. To continue pills, and use bromide enema to-night, preceded by one of warm water.

10th.—Vomits in the morning, but is improving in strength. Has taken for two days a pill night and morning, viz.: Oxalate of cerium, grs. xij.; leptandrin, ℈j.; ext. belladonna, grs. iij.; ext. taraxaci, q. s. M. F. pil. No. 12. Bowels moved naturally this morning. Can retain boiled eggs, but soup is rejected. Appears better, and her strength is improving. Continue pills.

19th.—Called in and found patient about the house, but vomits every morning, and sometimes at night. Has some fever towards morning. Bowels confined. Tongue slightly coated at the base. Can retain food in the morning and at noon, but rejects it at night. Is now taking lager beer at meals, in place of tea or coffee. Takes the pills, but with no effect as to taxation. To continue pills, with double the amount of leptandrin.

In this case there was more than the usual degree of debility from prostration. There appears to have been a general feverish condition which does not always obtain. The enemata produced decided good effects, and acted to quiet nervous irritability.

At this writing (April 12) the patient is better than usual under like circumstances, and has been about town for a month or two.

